# The Mechanical Interpretation of Ocular Response Analyzer Parameters

**DOI:** 10.1155/2019/5701236

**Published:** 2019-07-16

**Authors:** Xiao Qin, Mengyao Yu, Haixia Zhang, Xinyan Chen, Lin Li

**Affiliations:** ^1^Beijing Key Laboratory of Fundamental Research on Biomechanics in Clinical Application, Capital Medical University, Beijing 100069, China; ^2^School of Biomedical Engineering, Capital Medical University, Beijing 100069, China

## Abstract

**Purpose:**

Ocular Response Analyzer (ORA) is one of the most widely used devices in clinic, while the mechanical interpretations of parameters obtained from ORA have not been understood completely. The aim of this research is to explore the mechanical interpretation of ORA parameters.

**Methods:**

Rabbits aged 3-24 months were measured with ORA in vivo and corneal strips uniaxial tensile tests to get ORA parameters and corneal biomechanical parameters (corneal elastic modulus, relaxation time, and relaxation limit). The mechanical interpretation of ORA parameters was cognized preliminarily by analyzing the correlation between ORA parameters and corneal biomechanical parameters. On the other hand, finite element method was applied to simulate ORA measurements with different corneal biomechanical parameters to obtain quantitative relationship between ORA parameters and corneal biomechanical parameters further.

**Results:**

Biomechanical experimental results showed that Corneal Resistance Factor (CRF) was correlated with corneal elastic modulus and relaxation limit significantly, while the significant correlations between Corneal Hysteresis (CH) and corneal biomechanical parameters were not observed. Results of finite element analysis showed that both CH and CRF were correlated with corneal elastic modulus, relaxation limit, and relaxation time significantly. Besides, corneal elastic modulus was positively correlated with* upslop1* and* upslop2* and negatively correlated with* w2*.

**Conclusions:**

For all ORA parameters, CH, CRF, the upslope, and the width of the peaks are parameters which may reflect corneal elastic properties. It is viable to cognize mechanical interpretation of ORA parameters by the comparisons of the data from ORA and biomechanical tests of rabbits with different ages and the simulations of ORA based on finite element methods. Further studies are needed to confirm the mechanical interpretation.

## 1. Introduction

The cornea is the transparent soft tissue located in the outer layer of the eyeball and provides 70% ocular refractive power [1]. Unnormal corneal morphology may cause corneal diseases such as keratoconus and myopia. The change of corneal morphology is closely related to corneal biomechanical properties and will influence its refractive function [2]. Therefore, studying on corneal biomechanical properties has great significance in the prevention and diagnosis of corneal diseases such as keratoconus, individualized design, and prognosis of corneal refractive surgeries.

Similar to most biological tissues, corneal biomechanics include its anisotropic, nonlinear elastic properties and viscoelastic properties [3, 4]. Corneal strip tensile tests [5–7], corneal inflation tests [3, 8, 9], and indentation tests [10, 11] are three main methods to measure corneal biomechanical properties directly at present. According to the load-displacement data obtained by uniaxial tensile test, researchers can extract corneal elastic properties such as corneal elastic modulus. And from stress relaxation curve corneal viscoelastic properties can also be characterized [5, 7, 12]. However, corneal strip tensile experiments cannot be used in clinic directly. Ocular Response Analyzer (ORA) and Corneal Visualization Scheimpflug Technology (Corvis ST) are two of the most widely used devices for measurements of corneal biomechanical properties in clinic. The parameters provided by these devices such as Corneal Hysteresis (CH) and Corneal Resistance Factor (CRF) by ORA measurements and corneal deformation amplitude (DA) and first applanation time (1st A-time), etc., by Corvis measurements are descriptions of mechanical process of cornea under air-puff. These parameters are related to corneal biomechanics, intraocular pressure, and corneal geometrical parameters, so we call them corneal clinical biomechanical parameters. However, the biomechanical interpretation of these parameters has not reached a consensus yet, which causes the limitation of these devices in clinical applications.

As one knows some anatomical and histological properties of cornea change with the increase of age. There should also be some changes in corneal biomechanical parameters with the increase of age. A large number of clinical studies found that corneal clinical biomechanical parameters are correlated with age [13–17]. Elsheikh et al. [18] had studied the human corneal biomechanical properties aged between 50 and 95 years by corneal inflation tests and results showed an increase trend in corneal elastic modulus, while human cornea is too precious and few researches are related to the biomechanical properties of young people. Rabbit cornea is one of the most common used specimens to study corneal biomechanical properties [19–21] as rabbit eyes are similar to human eyes in size. In our previous paper, we had compared corneal biomechanical properties of 3-month rabbits and 7-month rabbits preliminarily, and the results showed difference between the two groups both in corneal elastic modulus and viscoelastic property [22]. So, in our last study, we proposed a possible way to study the relationship between corneal clinical biomechanical parameters and corneal traditional biomechanical parameters by the correlation analysis of rabbit corneal biomechanical parameters with different ages [23].

In this study, corneal strip tensile and stress relaxation experiments were carried out on rabbits with different ages to obtain corneal biomechanical properties. The results of the relationship between ORA parameters and age based on the same rabbits were reported in our last research [23]. Combining these results, we can recognize the mechanical interpretation of ORA parameters preliminarily. Besides, quantitative relationship between ORA parameters and corneal biomechanical parameters was obtained by finite element method. The results of this research will provide important information for the understanding of corneal clinical biomechanical parameters which play an important role in the diagnosis of corneal disease such as keratoconus and design of corneal refractive surgery.

## 2. Materials and Methods

### 2.1. Specimen Preparation

Twenty-Four New Zealand rabbits aged 3, 12, 18, and 24 months (6 for each age) with healthy eyes were enrolled in the study. All of the rabbits were provided by Animal Laboratory Center of Capital Medical University, and the experiments followed with the ARRIVE guidelines and NIH guidelines.

Central Corneal Thickness (CCT) was measured using Pachymeter SP 3000 before the animals were killed, and the result was showed in Table 1. Rabbits were anesthetized to death through intravenous injection of 20% urethane agent via rabbit auricular vein. The method of specimen preparation had been described in detail in Refs [24, 25] and here we summarized it as follows: a whole cornea with about 5.0mm sclera was assembled on a custom arched mold and was cut by a custom double-blade knife with width of 2.80mm. Strip length and width were measured with a Vernier caliper and the results were showed in Table 1. All of the experiments were carried out within 2 hours after the rabbits were killed.

### 2.2. Biomechanical Tests

**Figure 1:**
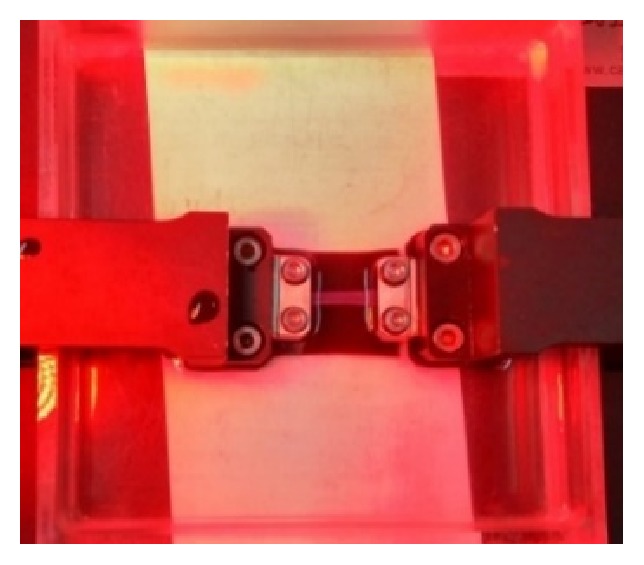
Clamping and water bath system of corneal strip.

All rabbits were anesthetized with 3% pentobarbital sodium (Merck, Germany) at a dose of 1ml/kg and measured with ORA in vivo for 4 times. The details of the methods for ORA measurements on rabbits have been reported in our previous study [23]. The screening condition was “WS>3.5.” After ORA measurements in vivo, corneal strips tensile experiments were performed on Care-IBTC-50 (In-situ Bi-directional Tension & Compression) Testing System (CARE Measurement & Control Corp., Tianjin, China) under room temperature and 9% saline bath environment. The corneal strip was fixed by a couple of metal grippers (Figure 1). Each of the specimens was subjected to a set of loading and unloading uniaxial tensions. After 6 cycles, the stress–strain curve became stable and specimens were regarded as preconditioned. The stress-strain test at a tensile rate of 0.02 mm/s was carried out afterwards. After a 5-minute recovery, all corneal strips were stretched at the rate of 0.5 mm/s till they became 125% of the original length and a 10-minute stress-relaxation test was performed. All uniaxial tensile tests were completed within 2 hours after the death of rabbits. There was no significant edema after the tests.

### 2.3. Mathematical and Statistical Analysis

The load-displacement data obtained from the corneal strip tensile tests were converted to stress-strain data by Eq (1):

(1) σ=FA0ε=λ2−12

In Eq (1) *σ* is Lagrange stress,* A*_0_ is the initial sectional area at the center point of the strips from the start, *ε* is Green strain, and *λ* = *L*/*L*_0_ is the extension ratio.* L*_0_ is the initial length of corneal strip.

**Figure 2:**
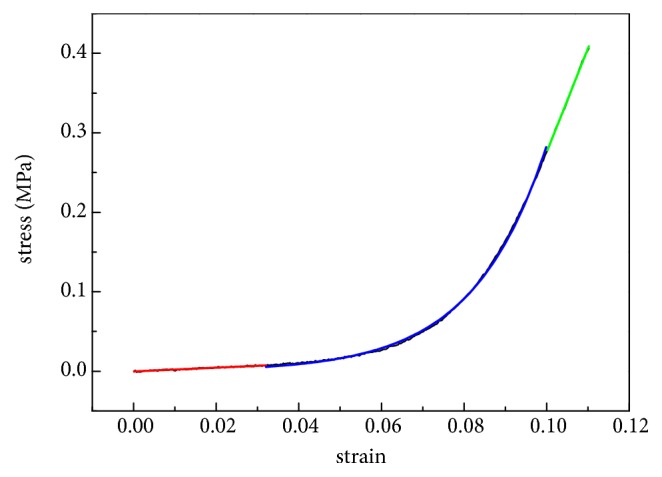
Stress-strain curve of corneal strip and regional division for the curve. The red, green, and blue line represent the physiological range, higher stress state, and nonlinear range, respectively.

The stress-strain curve was nearly linear under a lower stress of 0.015MPa-0.03MPa and a higher stress of 0.06MPa-0.1MPa (Figure 2) corresponding to 20mmHg-40mmHg and 80mmHg-130mmHg, respectively, according to Ref [6]. The slopes of stress-strain curve in these two stress ranges were calculated by linear fitting and named as physiological elastic modulus, denoted by* E*_1_, and elastic modulus under higher stress, denoted by* E*_2_. Researches have shown that exponential model can characterize the stress-strain relationship of soft tissue well [5, 7, 26, 27]; hereby we applied the following exponential model (Eq (2)) to fit the cornea stress-strain data under stress of 0.01MPa-0.1MPa:

(2) $$\begin{array}{c} \sigma =a\left(\;e^{b\varepsilon }-1\;\right) \end{array}$$

*a* and* b* are material constant parameters in Eq (2). From Eq (2), one gets that

(3) $$\begin{array}{c} E_{t}=\frac{d\sigma }{d\varepsilon }=abe^{b\varepsilon }=b\sigma +ab \end{array}$$

which gives the linear relation between corneal tangent modulus, *E*_*t*_ = d*σ*/d*ε*, and stress, *σ*. The following 3-term Prony model was chosen to fit the normalized stress relaxation data:

(4) Gt=1−a11−e−t/τ1−a21−e−t/τ2−a31−e−t/τ3

where* a*_1_,* τ*_1_,* a*_2_,* τ*_2_,* a*_3_,* τ*_3_ were constants,* G*(*t*)=*σ*(*t*)*/σ*_0_ is the normalized stress-relaxation function, *σ*(*t*) is the Lagrange stress, and* σ*_0_ is the initial stress. Corneal stress relaxation limit (*G*_*∞*_) was defined by the normalized stress value when *t* was *∞*. Stress relaxation time (*τ*) was defined as the time over which the stress was relaxed halfway between its initial and equilibrium value [28].

After obtaining above corneal biomechanical parameters, one-way ANOVA analysis was used to analyze the correlation between these parameters and age. Pearson's correlation was used to get the correlation between corneal clinical and traditional biomechanical parameters. All of the statistical analyses were produced on SPSS 21.0 and* p*<0.05 was considered statistically significant.

### 2.4. Explicit Finite Element Analysis of ORA Measurements

**Figure 3:**
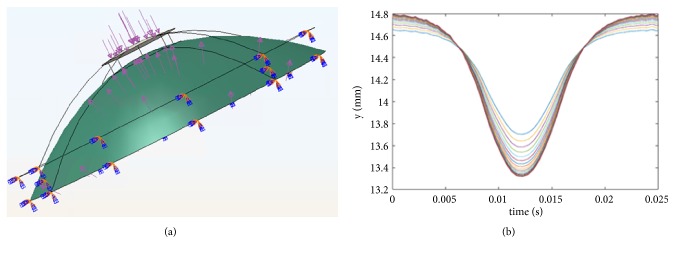
Finite element models of ORA measurements (a) and the output central corneal coordinate (b).

To obtain the correlation between ORA parameters and corneal biomechanical parameters quantitatively in further detail, we simulated ORA measurements with different corneal biomechanical parameters by finite element method. The geometrical model (Figure 3(a)) was built based on rabbit corneal geometrical image of optical coherence tomography (OCT). Cornea was hypothesized to be linear elastic and viscoelastic material. For each age, a set of corneal biomechanical parameters was selected random including corneal elastic modulus and 3-order Prony viscoelastic models' parameters. Poisson's ratio was set to be 0.49 due to incompressibility. Air puff force was applied on corneal apex as a 25-ms surface traction which was normal distribution with time and radius with an amplitude of 0.40MPa. The displacements of limbus are constrained (Figure 3(a)). Cornea was meshed with C3D8R mesh and explicit dynamic analysis was used to simulate the measurements. The finite element analysis was conducted on ABAQUS/Explicit. As corneal topography is measured at a specific intraocular pressure IOP and is distinct from the unloaded shape that would be obtained at an IOP of 0 mm Hg, the undeformed state was solved by a custom finite element model at first. The variation of central corneal coordinate along the air-puff force during the measurements was extracted (Figure 3(b)) to get the two applanation pressures (*P*_1_ and *P*_2_) and ORA parameters (CH and CRF) were calculated according to the following equations [29]:

(5) CH=P1−P2

(6) CRF=P1−0.7P2

As the difference between corneal biomechanical parameters in vivo and in vitro, corneal tangent modulus and parameters of the 3-order Prony model were adjusted to make the simulated ORA parameters have the same magnitude with experimental parameters. Besides, linear regression analysis was used to obtain the quantitative relation between ORA parameters and corneal biomechanical parameters preliminary.

## 3. Results

Table 1 gave the information of corneal strips including the thickness, length, and width of the corneal strips. From the table we can get that there was no significant difference in the length and width of corneal strips in different groups, while CCT changed significantly with the increase of age. The CCT was regarded as the initial thickness when calculating the stress.

### 3.1. Correlation between Corneal Elastic Properties and Age

**Figure 4:**
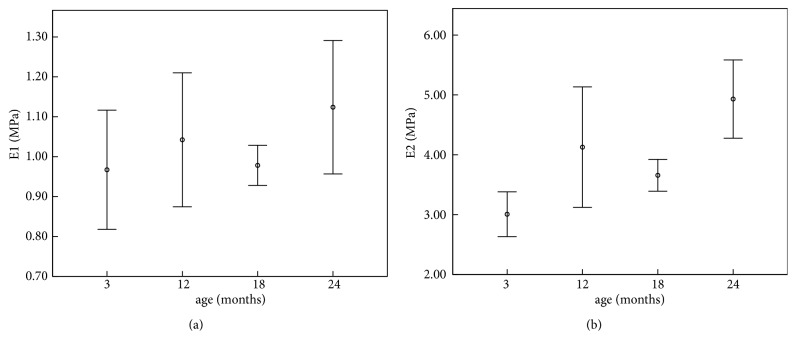
Variation of corneal elastic modulus with age (*E*_1_: physiological modulus;* E*_2_: elastic modulus under higher stress).

The results of the linear fitting and exponential fitting for stress-strain data were showed in Table 2, in which* E*_1_ and* E*_2_ represent corneal physiological elastic modulus and corneal elastic modulus under higher stress, respectively. Results of the one-way ANOVA analysis were listed in the last line of the table. From the results we can see that there was no significant variation in corneal physiological elastic modulus with the increase of age (*p*=0.256), while corneal elastic modulus under higher stress increased with the increase of age (*p*<0.001).  Figure 4 showed the variations of* E*_1_ and* E*_2_ with the increase of age.

**Figure 5:**
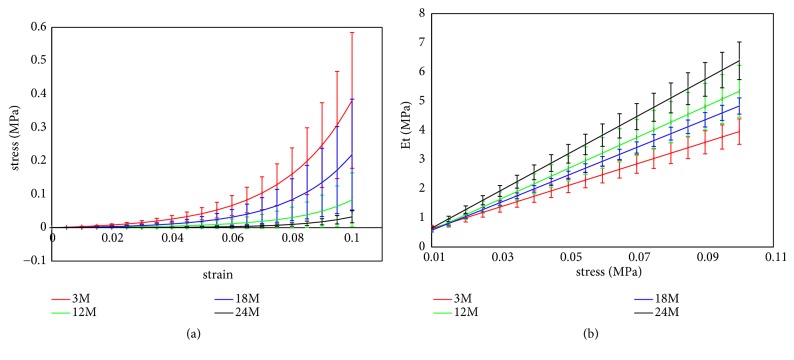
Stress-strain curve (a) and *e*_*t*_-stress curve (b) of corneal strips with different ages.

Corneal nonlinear elastic properties with different ages were showed in Table 2 and Figure 5. From Table 2 we can get that* a* and* b* in Eq (2) varied significantly with age (*p*<0.001). In Figure 5(a), stress-strain curves of corneal strips with different ages were provided. From the figure we can get that the stress was maximum in 24-month group and minimum in 3-month group when the cornea strips were under the same stress, and there was no significant difference between 12-month and 18-month groups. Corneal tangent modulus (*E*_t_) of corneal strips under different stresses was calculated according to the stress-strain data. And Figure 5(b) showed the *E*_t_-stress relations of corneal strips with different ages. Results showed that corneal tangent modulus was maximum in 24-month group and minimum in 3-month group when the corneal strips are under the same stress, and there was no significant difference between 12-month and 18-month groups; the slope of the *E*_t_-stress showed a similar variation trend. These variations were consistent to the strain variations when the corneal strips were under the same stress.

### 3.2. Correlation between Corneal Viscoelastic Properties and Age

The results of corneal stress relaxation were showed in Table 3. From the table we can get that there were significant differences in* τ*_1_ (*p*=0.008) and* a*_3_ (*p*<0.001), while no significant difference was found in other parameters in Eq (3). In the last 2 lines of the table we gave the relaxation limit (*G*_*∞*_) and relaxation time (*τ*). The corneal stress relaxation limit was found to have significant variation with age (*p*=0.045), but the significant correlation between relaxation time and age was not observed (*p*=0.224).

**Figure 6:**
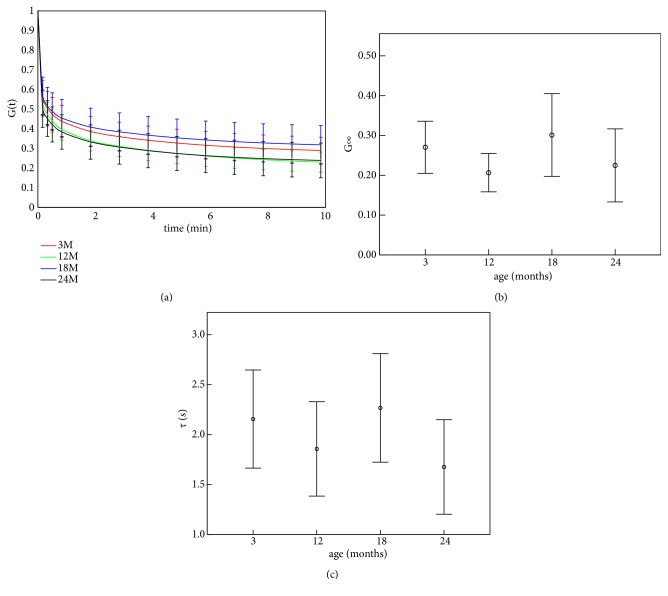
Stress relaxation curve (a) and corneal viscoelastic parameters (relaxation limit (b) and relaxation time (c)) of corneal strips with different ages.

Figure 6(a) shows the stress relaxation curves of corneal strips with different ages. From Table 3 we can get that corneal strips in 12-month and 24-month groups relaxed a little faster than in 3-month and 18-month groups. The variation of relaxation limit (Figure 6(b)) and relaxation time (*τ*) (Figure 6(c)) showed similar result: relaxation limit and relaxation time in 3-month and 18-momth groups were a little higher than in 12-month and 24-month groups.

### 3.3. Correlation between ORA Parameters and Corneal Biomechanical Parameters

**Figure 7:**
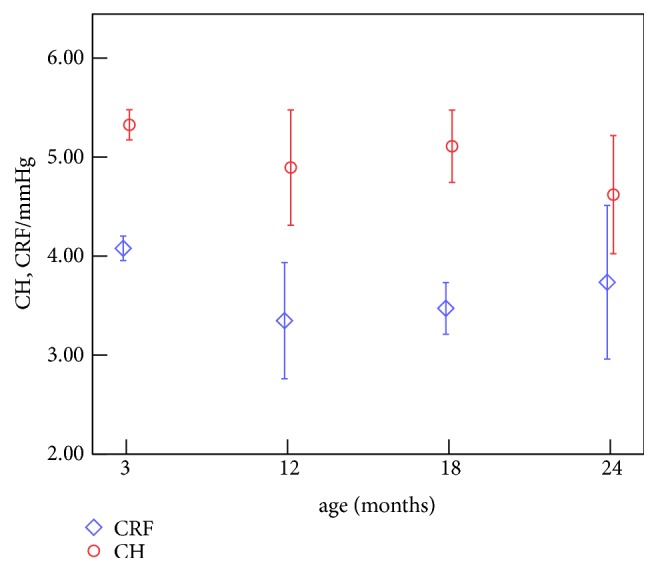
Age-related variations of ORA parameters.

Comparing the age-related variations of ORA parameters (Figure 7) [23] and corneal biomechanical parameters (Figures 4–6), we can find that CRF varied oppositely with increase of age compared to corneal tangent under higher stress (*E*_2_), and both CRF and CH showed similar trend with relaxation limit (*G*_*∞*_) and relaxation time (*τ*). Correlation analysis showed that CRF was positively correlated with* E*_2_ (*r*=0.490;* p*=0.007) and* b* (*r*=0.497;* p*=0.007), the slope of the linear relation between tangent modulus and stress, and negatively correlated with *G*_*∞*_ (*r*=0.374;* p*=0.045), while other correlations were not significant.

CH, CRF, and other 15 ORA waveform parameters (*p1area, p2area, p1area1, p2area2, uslope1, uslope2, uslope21, w1, w2, w11, w21, path1, path2, path11, *and* path21*) have been reported to have significant differences in different age groups in our last paper [23]. Comparing the variations of 15 ORA waveform parameters and corneal traditional biomechanical parameters with age, we can find that the variation of corneal elastic modulus was similar to those of the upslope of the peaks and the path length of the peaks, while opposite to those of the width of the peaks and the area under applanation peaks. Correlation analysis showed that corneal elastic modulus was positively correlated with* upslop1* (r=0.300; p=0.043) and* upslop2* (*r*=0.414;* p*=0.026) and negatively correlated with* w2* (*r*=0.322;* p*=0.039). Exponential fitting parameter* a* was negatively correlated with* upslop1* (r=0.336; p=0.035),* upslop2* (*r*=0.470;* p*=0.010), and* upslop21* (*r*=0.354;* p*=0.032) and positively correlated with* w2* (*r*=0.355;* p*=0.032). Exponential fitting parameter* b* was positively correlated with* upslop1* (r=0.376; p=0.030),* upslop2* (*r*=0.490;* p*=0.007), and* upslop21* (*r*=0.385;* p*=0.028) and negatively correlated with* w2* (*r*=0.324;* p*=0.037) and* w11* (*r*=0.343;* p*=0.034).

**Figure 8:**
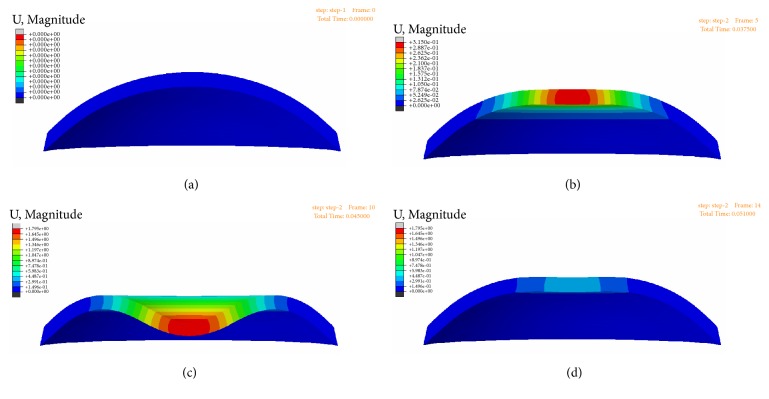
Cornea displacements distribution of the initial (a), the first applanation (b), the maximum indentation (c), and the second applanation state.

Explicit finite element method was used to explore the quantitative relation between ORA parameters and corneal biomechanical parameters.  Figure 8 exhibits the results of the simulation of ORA measurements using finite element method, and (a)–(d) represent the displacements distribution of the cornea at the initial state, the first applanation state, the maximum indentation state, and the second applanation state during the ORA test, respectively.

As the difference between corneal biomechanical parameters in vivo and in vitro, corneal tangent modulus and parameters of the 3-order Prony model were adjusted to make the simulated ORA parameters have the same magnitude with experimental parameters. Results showed that the simulated and experimental ORA parameters (CH and CRF) showed similar magnitude when corneal tangent modulus was set to be 1/3 of the corneal physiological tangent modulus and* τ*_1_,* τ*_2_,* τ*_3_ of the 3-order Prony model parameters was set to be 1/10 of the experimental results obtained from corneal strips extension experiments (Table 4).

To explore the relation between ORA parameters quantitatively, we maintain* a*_*2*_,* τ*_2_,* a*_3_,* τ*_3_ to be fixed and make* E*,* a*_1_,* τ*_1_ varied from 0.3-0.4MPa, 0.3-0.4, and 0.2-0.5s, respectively, when simulating ORA measurements. Corneal stress relaxation limit (*G*_*∞*_) and relaxation time (*τ*) can be calculated by Eq (4). The linear regression analysis results between ORA parameters and corneal biomechanical parameters are as follows:

(7) CH=6.596E−19.195G∞−0.670τ+9.234

(8) CRF=27.444E−37.424G∞−0.446τ−12.242

From Eq (7) and (8) we can find that both CH and CRF showed positive correlation with* E* and negative correlation with relaxation limit (*G*_*∞*_) and relaxation time* τ.* This was coincident with the relation between ORA parameters and corneal biomechanical parameters obtained by comparing these parameters of rabbits with different ages. These results showed that the correlation between ORA parameters and corneal biomechanical parameters got from this study was reliable.

## 4. Discussion

In this study, mechanical interpretation of ORA parameters was cognized preliminarily by comparing the variation of ORA parameters and corneal biomechanical parameters with age. And explicit finite element analysis of ORA measurements was used to get the quantitative relations further. CRF was found to vary oppositely with increase of age compared to corneal tangent under higher stress (*E*_2_), and both CRF and CH showed similar trend with relaxation limit (*G*_*∞*_) and relaxation time (*τ*). Explicit finite element analysis of ORA showed a similar correlation between ORA parameters and corneal biomechanical parameters. The results of the correlation between ORA parameters and corneal biomechanical parameters will help us to get better applications in clinic from ORA data of patients. Besides, the results of corneal biomechanical properties of rabbit with different ages showed corneal biomechanical parameters varied with the increase of age. Considering that rabbit cornea is one of the most commonly used corneal specimens in researches, the results of this study are very useful and important to the studies on rabbit cornea, such as the studies of biomechanical properties of rabbit cornea after laser in situ Keratomileusis with different repair time [30] and studies on the biomechanical responses to corneal cross-linking in rabbits [31].

Exponential model [5, 7, 26, 27] and Prony model [32, 33] have been found to well characterize corneal stress-strain data and stress relaxation data, respectively. From Table 2 and Table 3, the goodness-of-fit (R^2^) for the two linear fittings, exponential fitting, and Prony model fitting was larger than 0.92, 0.98, 0.99, and 0.99, respectively, which indicated that our fitting methods were effective. Results of corneal inflating tests also showed a linear corneal apex increase with pressure of 15-30 mmHg [2], and matrix-regulated phase was suggested in this range in which the biomechanical behavior is mainly dominated by the corneal matrix. According to Ref [6], this pressure range is corresponding to 0.015MPa-0.03MPa on corneal strips. Therefore, it is reasonable to regard corneal tangent modulus under stress 0.015MPa-0.03MPa as physiological modulus. The range of corneal physiological modulus (*E*_1_) was 0.7-1.5MPa in this study, the ranges of parameters of exponential model (*a* and* b*) in Eq (2) were 0-0.02MPa and 27-92, respectively, and the range of relaxation limit (*G*_∞_) was 0.13-0.45. These results are coincident with these parameters reported by Wang (*E*_1_=1.60±0.38MPa) [30], Hatami-Marbini (*a*=0.0077MPa-0.0083MPa;* b*=50-80) [5, 12], Elsheikh (*a*=0.042MPa-0.342MPa;* b*=30-31 [8];* G*_∞_=0.3-0.5 for porcine cornea and 0.6-0.8 for human cornea [3]), and others at the magnitude.

From Table 2 and Figures 4 and 5, there was no significant variation in corneal physiological modulus with the increase of age, while corneal elastic modulus under higher stress showed an increase trend with the increase of age; the nonlinear elastic properties, i.e., the slope of tangent modulus-stress curve (parameter* b*), increased with age. Our previous study on the rabbits aged 3 and 7 months [22] showed that the tangent modulus increased slightly with the increase of age, which was coincident with the results of modulus under higher stress in this study. Another study involving rabbits aged 4-48 months [14] showed a similar trend in corneal elastic modulus. Results of inflating tests of 50-95-year-old human cornea [18] also showed an increase trend in corneal elastic modulus and parameter* b*. Anderson et al. had proposed a hypothesis that corneal matrix contributes most for the biomechanical properties under low stress and fibril layers contribute most under high stress [2]. Based on this hypothesis, we may speculate that biomechanical properties of corneal fibril layer vary significantly with age while there are no significant variations in biomechanical properties of corneal matrix with age. Table 3 and Figure 6 showed that corneal relaxation limit and relaxation time decrease from 18 to 24 months and increase from 12 to 18 months. Results of 3 months and 7 months showed that relaxation rate increases from 3 to 7 months, which is coincident with the trend from 3 months to 12 months in this study [22]. Corneal creep rate was found to decrease slightly from middle rabbits to old rabbits [14], which is also coincident with our results from 18 months to 24 months. According to the relationship between the ages of rabbit and human, rabbits aged 3, 12, 18, and 24 months roughly correspond to 5, 18, 25, and 35 years of human, respectively [23]. Based on this age correspondence and the assumption that corneal development process of human and rabbit is consistent, we may infer that corneal elastic modulus under higher stress increases gradually, and corneal relaxation limit and relaxation time decrease significantly from 18 years old to 25 years old and increase significantly after 25 years old. Some corneal diseases such myopia and keratoconus may be correlated to corneal biomechanical properties, and the minimum elastic modulus at 5 years and increase of corneal relaxation from 5 to 18 years may explain why myopia and keratoconus are often observed in adolescence from a biomechanical point of view. As corneal stress relaxation was smaller in 18 years and 35 years and corneal elastic modulus in 18 years is still not large enough, corneal refractive surgery should better be operated after 18 years old to avoid postoperative corneal ectasia.

From Figures 4–7 we can find that CRF varied oppositely with increase of age compared to corneal tangent under higher stress (*E*_2_), and both CRF and CH showed similar trend with corneal stress relaxation limit (*G*_∞_) and relaxation time (*τ*). While there are few researches that reported the relationships between ORA parameters and corneal traditional biomechanical parameters, the positive correlation between CRF and corneal tangent modulus is consistent with the following researches: keratoconus patients have lower CH and CRF values [34] and reduction in elastic modulus [35]. Researches have reported that CRF values decreased [36] and corneal relaxation limit values increased after LASIK [24], which is consistent with the negative correlation between CRF and* G*_∞_. Besides, explicit finite element analyses of ORA measurements also show positive correlations between ORA parameters (CH, CRF) and corneal tangent modulus* E* and negative correlations between ORA parameters and relaxation limit (*G*_∞_) and relaxation time (*τ*). These results indicated that the correlation between ORA parameters and corneal biomechanical parameters of this study was reliable. Keratoconus patients that demonstrate a decrease in the Young modulus [37] and determine corneal elastic modulus in vivo may provide an effective approach for early diagnosis of Keratoconus. Although corneal elastic was not obtained directly in this study, Eq (7) and (8) give a quantitative relation to evaluate corneal elastic modulus. If we assume that relaxation time (*τ*) was constant and set to be 2s (Table 3), for example, then one can get the corneal elastic modulus and relaxation limit from ORA parameters (CH, CRF) by Eq (7) and (8), which gives that both corneal elastic modulus and relaxation limit linearly depend on CH (negatively) and CRF (positively). This may help ophthalmologists and researchers to explore a new method to diagnose early keratoconus based on ORA measurements. Correlation analysis between ORA waveform parameters and corneal biomechanical parameters showed that the upslope of the peaks and the width of the peaks are parameters which may reflect corneal elastic properties. Combining with the results that the variation of CRF was similar to those of the width of the peaks in the applanation curve, while it was opposite to those of the upslope of the peaks, we guess that the width of the peaks in the applanation curve, the upslope of the peaks, and the path length of the peak may be related to the stiffness of the cornea because these parameters reflect corneal deformability under external force, while the mechanical interpretation of ORA waveform parameters needs abundant data to acquire more accurate relation between these parameters and corneal biomechanical parameters.

The limitations of this study lied in 2 aspects. Cornea is an anisotropic material and its biomechanical properties should be characterized by the biomechanical properties of corneal strips in different directions (such radial and circumferential corneal strips). As it is difficult to obtain circumferential corneal strips for tensile tests, we selected corneal strips in nasal-temporal direction and superior-inferior direction. But no significant difference was found between the two directions. Tests on more directions may be needed to characterize corneal biomechanical properties more comprehensively. Another limitation of this study is that when we simulate ORA measurements, we calculated CH and CRF according to Eq (5) and (6), which may be not the real relation of ORA parameters and* P*_1_,* P*_2_. While the relationship has not been determined at present, researches have reported that experimental CH and CRF are linear positive correlation with the calculated CH and CRF [29] and our aim was to find the correlation between ORA parameters and corneal biomechanical parameters qualitatively. Therefore, it is feasible to calculate CH and CRF by Eq (5) and (6).

## 5. Conclusions

Mechanical interpretation of ORA parameters was cognized preliminarily by comparing the variation of ORA parameters and corneal biomechanical parameters with age. Explicit finite element analysis of ORA showed a similar correlation between ORA parameters and corneal biomechanical parameters. Both CRF and CH are positively linearly related to corneal elastic modulus and negatively linearly depend on relaxation limit and relaxation time and relaxation limit (*G*_*∞*_), which indicated that our method to study the mechanical interpretation of ORA parameters is viable. The results of the study will be expected to get better applications in clinic from ORA data from patients. Besides, the method used in this study for cognizing the mechanical interpretation of ORA parameters can also be used for getting the mechanical interpretation of parameters obtained from other clinical devices such as Corvis ST.

## Figures and Tables

**Table 1:** Information of corneal strips with different ages.

Age/months	CCT/*μ*m	Length/mm	Width/mm
3	349±14	15.17±1.13	3.21±0.14
12	375±26	15.27±2.13	3.33±0.10
18	389±20	16.25±0.94	3.42±0.18
24	373±28	16.28±1.29	3.38±0.12

**Table 2:** Linear and exponential fitting results of the stress-strain data.

Age/month	Linear fitting				Exponential fitting		
	*E*_1_/MPa	R^2^	*E*_2_/MPa	R^2^	*a*/MPa	*b*	R^2^
3	0.97±0.24	0.929±0.027	3.01±0.59	0.988±0.005	0.0070±0.0048	37±8	0.998±0.001
12	1.04±0.22	0.939±0.020	4.13±1.31	0.989±0.002	0.0013±0.0020	53±18	0.998±0.001
18	0.978±0.079	0.972±0.008	3.66±0.42	0.993±0.002	0.0021±0.0020	47±6	0.999±0.001
24	1.124±0.263	0.949±0.017	4.93±1.03	0.988±0.017	0.0002±0.0001	64±13	0.998±0.002
*p*	0.256		<0.001*∗*		<0.001*∗*	<0.001*∗*	

*E*_1_: physiological elastic modulus; *E*_2_: elastic modulus under higher stress.

*∗*: there was significant difference in different groups.

**Table 3:** Results of the stress relaxation with different ages.

Age/months	3	12	18	24	*p*
*a*_1_	0.40±0.09	0.42±0.07	0.39±0.11	0.45±0.10	0.499
*τ*_1_/s	3.07±0.39	2.89±0.34	3.01±0.25	2.63±0.27	0.008*∗*
*a*_2_	0.16±0.03	0.18±0.04	0.15±0.02	0.16±0.02	0.155
*τ*_2_/s	61±58	29±13	86±84	55±47	0.282
*a*_3_	0.16±0.02	0.19±0.01	0.15±0.02	0.17±0.01	<0.001*∗*
*τ*_3_/s	238±117	286±139	207±141	209±95	0.344
*R* ^2^	0.995±0.001	0.999±0.001	0.999±0.001	0.999±0.001	
*G*_∞_	0.27±0.07	0.21±0.05	0.30±0.10	0.22±0.09	0.045*∗*
*τ*/s	2.15±0.73	1.86±0.61	2.27±0.86	1.68±0.74	0.224

*G*_*∞*_: relaxation limit; *τ*: relaxation time.

*∗*: there was significant difference in different groups.

**Table 4:** Results of finite element analysis of ORA measurements.

age/months	3	12	18	24
Experimental CH/mmHg	5.32	4.86	5.13	4.53
Simulated CH/mmHg	6.10	5.98	6.04	5.64
Experimental CRF/mmHg	4.49	3.64	4.12	3.41
Simulated CRF/mmHg	6.25	4.83	5.70	4.31

## Data Availability

The data used to support the findings of this study are available from the corresponding author upon request.
