# Corrigendum to “The Mechanical Interpretation of Ocular Response Analyzer Parameters”

**DOI:** 10.1155/bmri/9781847

**Published:** 2025-09-04

X. Qin, M. Yu, H. Zhang, X. Chen, and L. Li, “The Mechanical Interpretation of Ocular Response Analyzer Parameters,” *BioMed Research International* 2019 (2019): 5701236, https://doi.org/10.1155/2019/5701236

In the article, there is an error in Figure 8 introduced by the authors during the preparation of the figure. The correct Figure 8 is shown below:

Figure 8(a) Cornea displacement distribution of the initial, (b) the first applanation, (c) the maximum indentation, (d) and the second applanation states. The colorbars of (b) and (d) are the same because of the similar applanation states.

**Figure:**
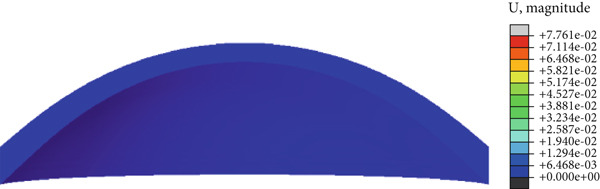
(a)

**Figure:**
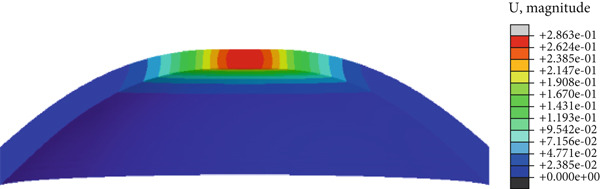
(b)

**Figure:**
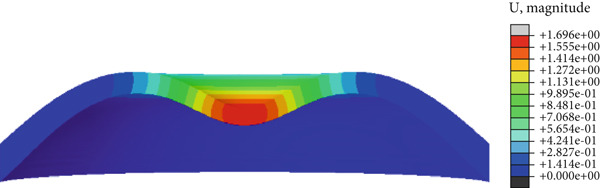
(c)

**Figure:**
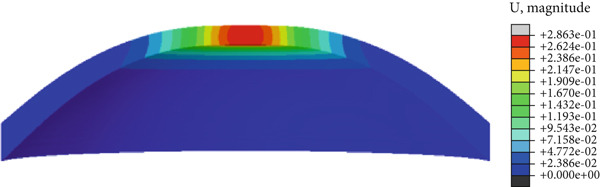
(d)

Additionally, there is an error in Equation (6). The correct Equation (6) is shown below, where *k*_1_ and *k*_2_ are constants.

(6) CRF=k1P1−0.7P2+k2.

We apologize for these errors.

